# F-box proteins and gastric cancer: an update from functional and regulatory mechanism to therapeutic clinical prospects

**DOI:** 10.7150/ijms.91584

**Published:** 2024-06-03

**Authors:** Yanzhen Yang, Qu Xie, Can Hu, Jingli Xu, Lei Chen, Yuan Li, Cong Luo

**Affiliations:** 1 Postgraduate Training Base Alliance of Wenzhou Medical University (Zhejiang Cancer Hospital), Hangzhou, Zhejiang, 310022, China.; 2 Zhejiang Cancer Hospital, Hangzhou, Zhejiang, 310005, China.

**Keywords:** F-box proteins, Gastric cancer, Mechanism, Non-coding RNA, Chemoresistance

## Abstract

Gastric cancer (GC) is a prevalent malignancy characterized by significant morbidity and mortality, yet its underlying pathogenesis remains elusive. The etiology of GC is multifaceted, involving the activation of oncogenes and the inactivation of antioncogenes. The ubiquitin-proteasome system (UPS), responsible for protein degradation and the regulation of physiological and pathological processes, emerges as a pivotal player in GC development. Specifically, the F-box protein (FBP), an integral component of the SKP1-Cullin1-F-box protein (SCF) E3 ligase complex within the UPS, has garnered attention for its prominent role in carcinogenesis, tumor progression, and drug resistance. Dysregulation of several FBPs has recently been observed in GC, underscoring their significance in disease progression. This comprehensive review aims to elucidate the distinctive characteristics of FBPs involved in GC, encompassing their impact on cell proliferation, apoptosis, invasive metastasis, and chemoresistance. Furthermore, we delve into the emerging role of FBPs as downstream target proteins of non-coding RNAs(ncRNAs) in the regulation of gastric carcinogenesis, outlining the potential utility of FBPs as direct therapeutic targets or advanced therapies for GC.

## Introduction

Gastric cancer (GC) poses a significant global health challenge, imposing a substantial burden on public health. In 2020 alone, GC accounted for over 1 million new cases and caused more than 768,000 deaths, ranking it as the third leading cause of cancer-related mortality worldwide 1, 2. Notably, the incidence and mortality rates of GC are highest in East Asian countries, particularly in China, where GC has become the second leading cause of cancer-related deaths 3-5, this trend can be attributed to dietary habits and environmental factors 6. Surgical resection is the preferred treatment approach for early-stage GC patients (stages I to III) 7, 8. However, due to the often-asymptomatic progression in the early stages, diagnosis is frequently delayed, resulting in the majority of GC cases being diagnosed at advanced stages (>80%). Consequently, many patients miss the opportunity for surgery, leading to poor prognosis and increased mortality 9, 10. For patients who are ineligible for surgical resection or have advanced metastases, the combination of chemotherapy, targeted therapy, and immunotherapy represents the primary treatment option. However, treatment failure is common due to resistance and limited efficacy 11, 12. Despite advancements in therapeutic development, the overall 5-year survival rate for advanced GC patients remains below 40% 13. Thus, there is an urgent need for novel treatment strategies for GC. Recent studies have indicated that the ubiquitin-proteasome system-mediated degradation of oncogenes and oncoproteins plays a crucial role in the development and progression of GC.

**Figure 1:**
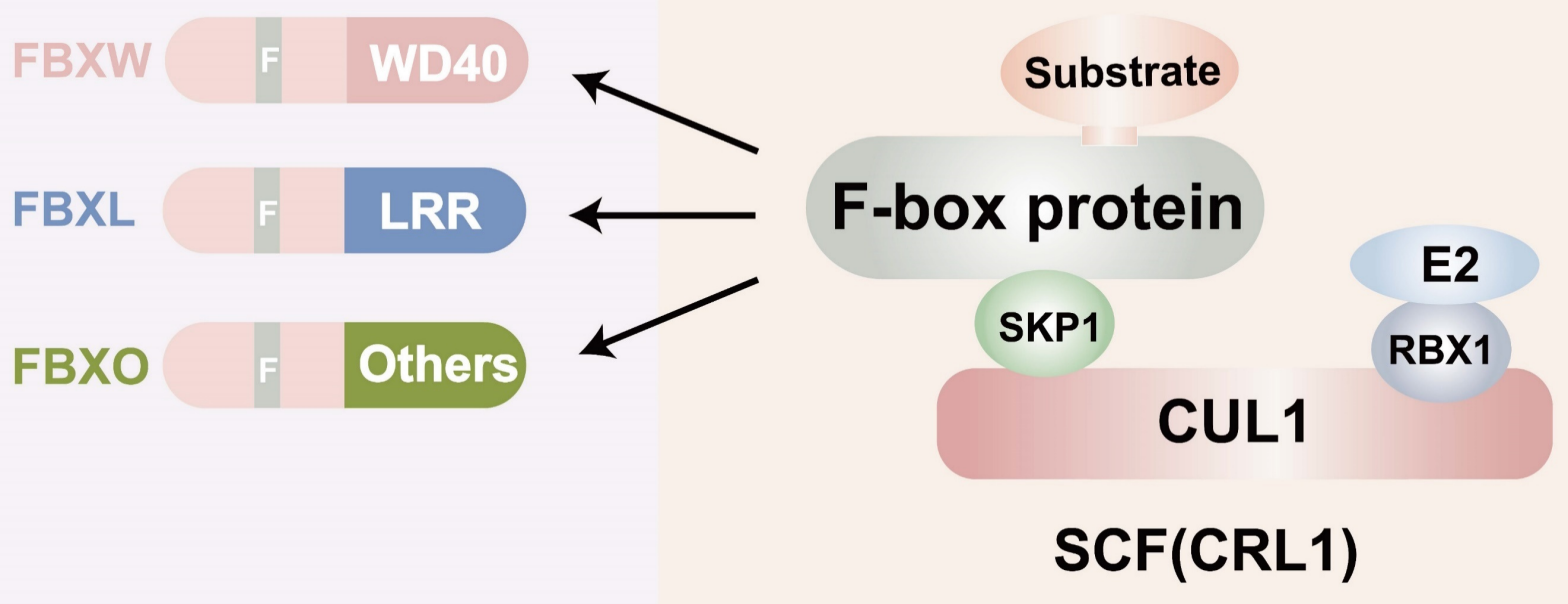
Structure of the F-box proteins. CUL: Cullin; SCF: Skp1-Cullin-F box complexes; CRL1: cullin-RING ubiquitin ligase1; RBX1; RING-domain-containing partner.

F-box proteins, though structurally simple, exhibit exceptional functional. They complexity consist of three main domains: the N-terminal F-box domain, the middle linker domain, and the C-terminal functional domain (**Figure 1**). The F-box domain, approximately 50 amino acids in length, is highly conserved and plays a critical role in identifying FBPs. It interacts with the Skp1 protein to form the foundation of the SCF (Skp1-Cullin1-F-box protein) complex 14. The linker domain, generally composed of about 30 amino acids, acts as a bridge between the F-box domain and the C-terminal functional structure. The size and function of the C-terminal domain vary across different F-box proteins and can be categorized into various types, such as RING finger structures with ubiquitin ligase activity and WD40 repeat structures involved in protein degradation 15, 16. Furthermore, the functional diversity of F-box proteins and their interactions constitute significant structural features. F-box proteins participate in numerous vital physiological and pathological processes in organisms, including cell cycle regulation, cell proliferation, gene expression and regulation, apoptosis, and signal transduction 17. They engage in a wide array of protein-protein interactions, including interactions with phosphatases, regulation of activity through second messengers, and involvement in protein subcellular localization 18-20. Given that each F-box protein has multiple substrates, determining whether they exert anti-tumor or tumor-promoting effects is a complex question and may depend on the cellular context. A large body of data now suggests that FBPs have oncogenic or tumor suppressor activity 21. Some of them, such as FBXW7, are mutated or show high-frequency expression deregulation in a large number of human malignant tumors, suggesting a key role in cancer development or progression. For example, SKP2 is overexpressed in breast, prostate, colorectal, and pancreatic cancers as well as lymphoma, melanoma, and nasopharyngeal carcinoma, and is highly correlated with poor tumor prognosis 22-24. Furthermore, Lu *et al.* found that FBXO44 regulates BRCA1 gene stability in breast cancer 25. This reflects the important role of FBPs in cancer development. In this paper we will focus on the functional mechanism of FBPs in GC. Several F-box protein members, such as FBXO31, FBXL7, FBXO9, FBXO44, and FBXW11, have been found to be aberrantly expressed in human GC, suggesting their close association with gastric carcinogenesis and development. Additionally, certain F-box subfamilies, such as SKP2 and β-TrCP, are implicated in GC development and are considered prognostic factors with potential value for GC treatment.

This review paper aims to elucidate the signaling pathways, underlying mechanisms, and functional roles of FBPs in GC. Firstly, we comprehensively illustrate the stimulatory effects exerted by FBPs on proliferation, apoptosis, and invasive metastasis within the context of GC. Furthermore, we highlight their significant involvement in the development of chemoresistance. Secondly, we provide a comprehensive summary of the pivotal molecular mechanisms through which FBPs function as downstream target proteins of ncRNAs to orchestrate the process of gastric carcinogenesis. Lastly, we engage in a thorough discussion regarding the potential utility of targeting FBPs directly or employing them as advanced therapeutic strategies for the management of GC.

## 1. Overview of F-box proteins

### 1.1 The ubiquitin-proteasome system and the F-box proteins

**Figure 2:**
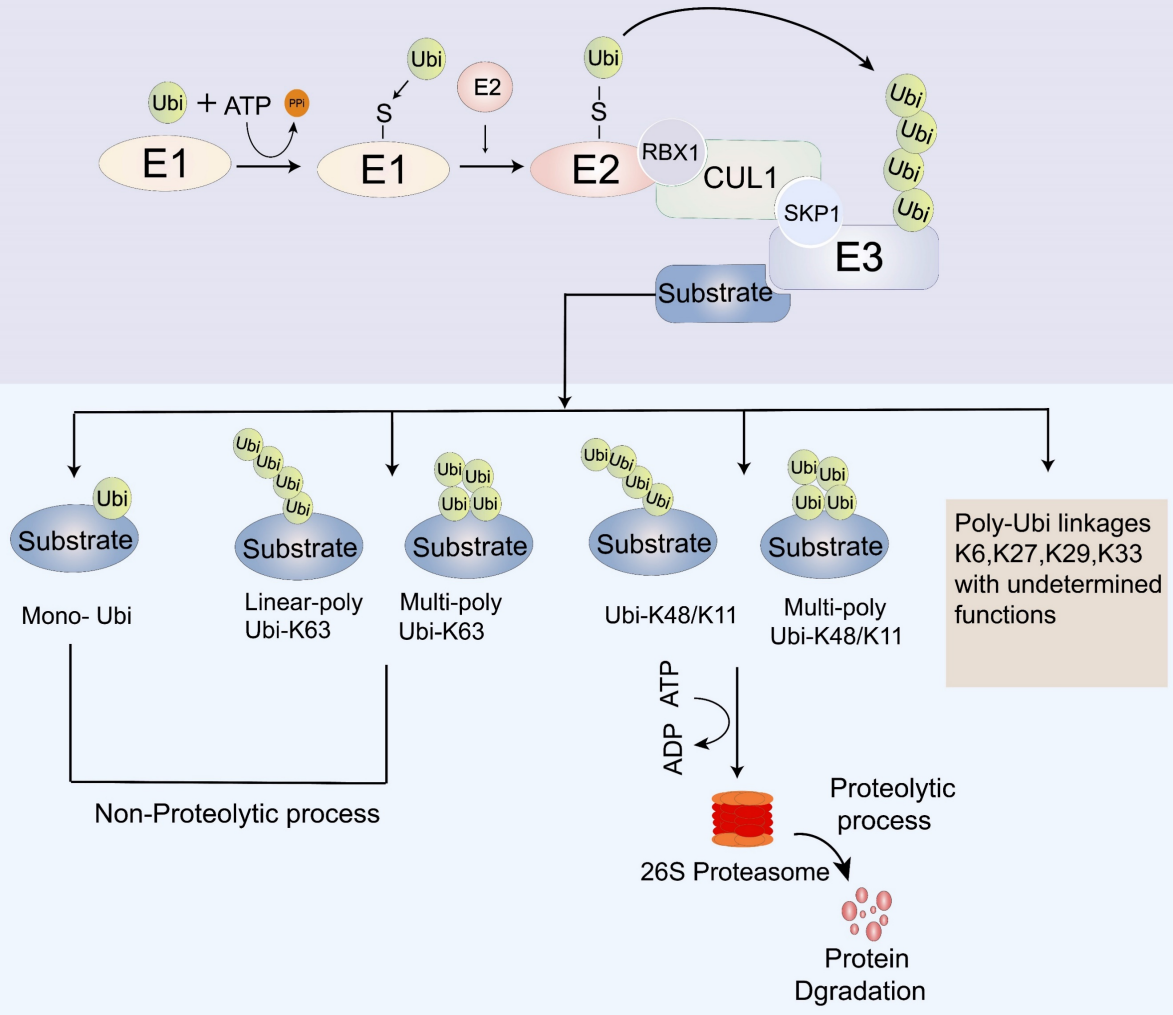
The process of ubiquitination modification. E1: ubiquitin-activating enzymes; E2: ubiquitin-conjugating enzymes; E3: ubiquitin ligases; Ubi: Ubiquitin; CUL: Cullin; SCF: Skp1-Cullin-F box complexes; CRL1: cullin-RING ubiquitin ligase1; RBX1; RING-domain-containing partner.

Cancer development entails the transformation of normal cells into cancer cells in response to abnormal cellular stimuli. This process is tightly regulated by the transcription, translation, post-translational modification, and degradation of key regulatory proteins, which play a vital role in maintaining cellular homeostasis. Within cells, two main systems are responsible for protein degradation: the autophagic lysosomal system and the ubiquitin-proteasome system (UPS) 26. The lysosomal pathway facilitates the degradation of extracellular proteins introduced into the cell through endocytosis or cytokinesis, while the UPS controls the degradation of intracellular proteins 27. Ubiquitin modification, a post-translational modification, involves a series of essential components, including ubiquitin-activating enzymes (E1s), ubiquitin-conjugating enzymes (E2s), ubiquitin ligases (E3s), deubiquitinating enzymes (DUBs), and the 26S proteasome 28-31. The UPS carries out its biological function through a cascade of three enzymatic reactions catalyzed by the ubiquitin-activating enzyme E1, the ubiquitin-conjugating enzyme E2, and the ubiquitin-protein E3 ligase 28. Initially, E1s activate ubiquitin (Ub) and form a thioester bond between the sulfhydryl group of the active cysteine residue of the E1s and the carboxyl group of Ub, which is then delivered to the E2s. Ultimately, through the synergy between substrate binding, E3 ligase, and ubiquitin-charged E2, polyubiquitinated target proteins are produced for degradation in the 26S proteasome 31-36 (**Figure 2**).

E3 ligase, a crucial component of the ubiquitination cascade, determines substrate specificity for ubiquitination and subsequent degradation. Based on their structural characteristics, E3 ligases can be primarily classified into three categories: RING (really interesting new gene, including U-box E3 with similar topology), HECT (homologous to E6AP C-terminus), and RBR (RING-in-between-RING). The largest family among them is the cullin-RING E3 ligase (CRL) complex family, which comprises eight members (CRL1, 2, 3, 4A, B, 5, 7, and 9) 37, 38. These ligases are recognized as key regulators of several cellular processes, including cell cycle progression, such as S-phase entry and G2/M-phase exit. Among them, CRL1, also known as the SCF E3 ligase complex, is the best-characterized member of the E3 ligase family 39.

**Figure 3:**
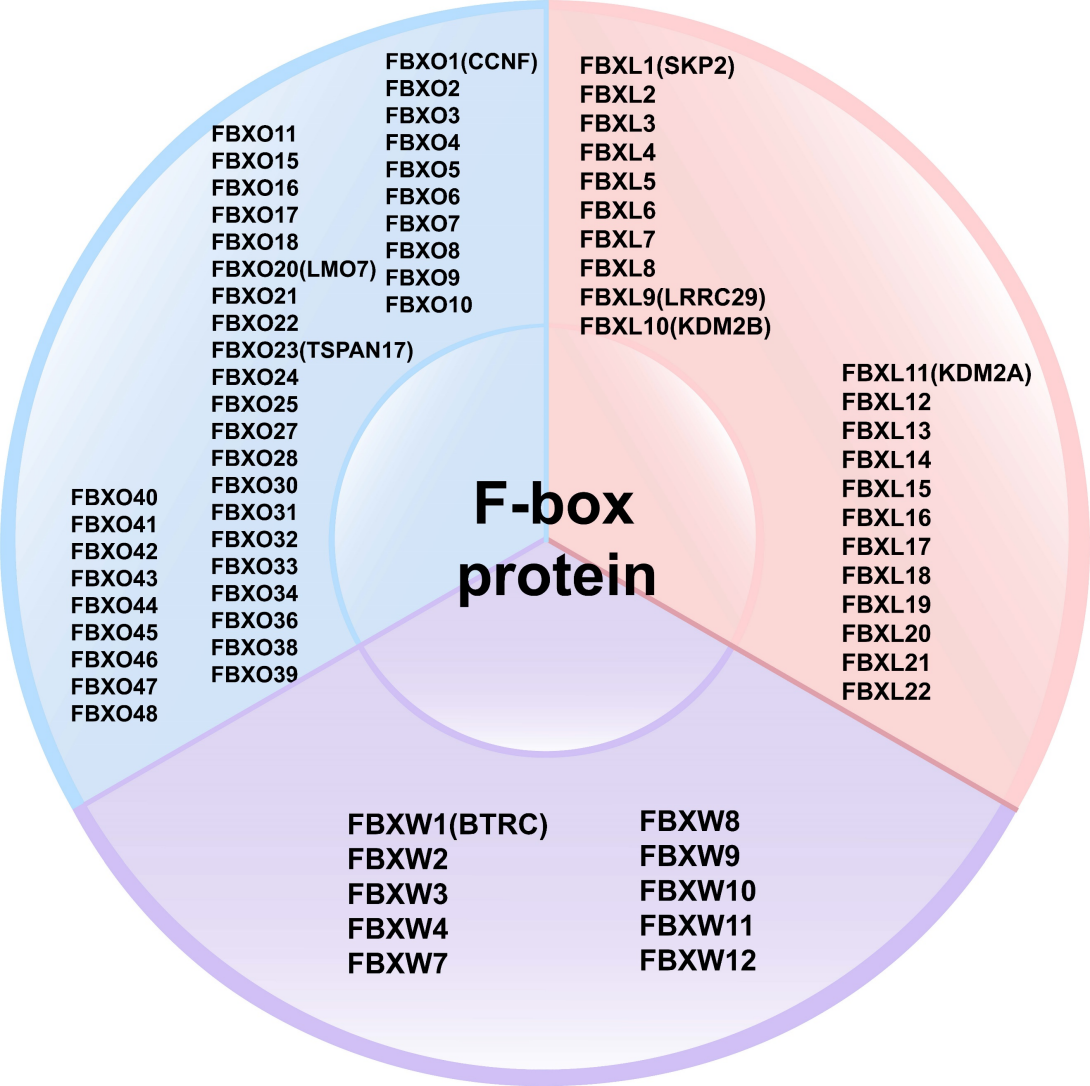
Classification of F-box proteins: 10 FBXWs with WD40 repeat domains, 22 FBXLs containing leucine-rich repeats, and the remaining 37 FBXOs with domains not observed in FBXWs and FBXLs subtypes.

FBPs can be classified into three subclasses based on the presence of specific substrate recognition structural domains: 10 FBXWs with WD40 repeat domains, 22 FBXLs containing leucine-rich repeats, and the remaining 37 FBXOs with domains not observed in FBXWs and FBXLs subtypes 40-42 (**Figure 3**). FBPs can bind to other proteins to form an E3 ubiquitin ligase complex that mediates the ubiquitination and degradation of the target protein 20, 39. Currently, several FBPs have been implicated in cell cycle progression, drug resistance, cell growth, repair, and differentiation 43-45.

### 1.2 Cell cycle and the F-box proteins

FBPs are actively involved in the regulation of the UPS and can promote the ubiquitination and degradation of target proteins, thereby influencing the cell cycle. Some studies have found the destabilization of E2F1 by the SCF^SKP2^ ligase may be important to limit its activity in S and G2 phases of the cell cycle44. Further studies revealed that SKP2 promotes not only the G1/S transition but also the G2/M transition through targeted protein hydrolysis of p27 and p21^46^, ^47^. In breast cancer cells and melanoma, elevated levels of FBXO31 protein induce degradation of cell cycle protein D1, leading to cell cycle arrest in G1^42^, Similarly β-TRCP1/2 proteins can regulate cell cycle progression by modulating CDK1 kinase activity 44. FBXL2 by facilitating the ubiquitin-mediated degradation of crucial cell cycle regulators including cyclin D2, cyclin D3 and Aurora B. D-type cyclins partner with CDK4 and CDK6 to drive G1-to-S cell-cycle progression 48. This suggests that FBPs have important regulatory roles in various processes of the tumor cell cycle. In GC, FBPs play a significant role in cell cycle regulation through two major mechanisms: Firstly, by regulating the ubiquitination of cell cycle regulatory molecules, FBPs can recognize, bind, and ubiquitinate a series of relevant proteins, thereby participating in cell cycle regulation 49, 50. For instance, F-box proteins regulate the degradation and stabilization of proteins such as the CDK inhibitors p27 51, p21 52 and CDC6^53^, which in turn control the cell cycle. Secondly, F-box proteins participate in signaling pathways to regulate the cell cycle. They serve as crucial regulatory molecules in various signaling pathways, such as Wnt 54, NF-κB 55 and HIF-1α pathway 56 by mediating the degradation of key molecules through ubiquitination, thereby influencing the cell cycle and other biological processes. In summary, FBPs play a pivotal role in cell cycle regulation by targeting key molecules and signaling pathways.

### 1.3 Therapeutic drug targets and the F-box proteins

A growing body of evidence now supports the promising development of FBPs for tumor therapy. A universal proteasome inhibitor velcade (bortezomib) for the treatment of multiple myeloma, targeting the UPS for tumor therapeutic purposes proved to be a promising approach 57. However, non-selective inhibition of protein degradation causes undesirable side effects, limiting the use of this approach. Since the substrate specificity of UPS is achieved by E3 ligases, such proteins offer new avenues for tumor therapy. Interestingly, some studies found that FBPs can impact the metabolism and clearance rate of drugs by regulating the expression and activity of drug-metabolizing enzymes, thereby modulating the efficacy and toxicity of drugs and enhancing the effectiveness and safety of drug therapy 58, 59. Furthermore FBPs also can influence the therapeutic effects of drugs by modulating the degradation rate of target proteins 60. For example, Tang *et al.* demonstrated that β-TrCP-deficient cells are more sensitive to various anticancer drugs (e.g., adriamycin, tamoxifen, and paclitaxel) on human mammary tumor cells 61. Several studies have demonstrated that targeting FBPs can suppress the growth and metastasis of tumor cells 28. Yang *et al.* developed a chemical genetics approach to overexpress SKP2 to anti-proliferative activity by restoring p27(Kip1) in prostate cancer cells^62^. Similarly, Wu *et al.* screened and identified a small molecule inhibitor specific for SCF-SKP2 activity, which selectively inhibits SKP2-mediated p27 degradation in cancer cells by reducing p27 binding via key compound-receptor contacts, thereby inhibiting tumor growth 63. However, most of the current inhibitors specific for FBPs remain in preclinical studies 60. It's worth mentioning that, agonists targeting F-box proteins are being investigated for the treatment of neurological diseases, cardiovascular diseases, and other conditions 28, 64. Therefore, the development of therapeutic agents targeting FBPs holds great potential for delivering excellent therapeutic outcomes in cancer clinical treatment.

## 2. Expression of the F-box proteins in gastric cancer

A genomic profiling revealed that FBXW7 mutations were observed in 9.2%-18.5% of GC tumors and 4.7% in IM (Intestinal Metaplasia), and suggested that FBXW7 mutations in IM are likely to functionally contribute to IM and GC development 65. The expressions of FBPs are generally decreased in GC, including FBXW7, FBXL2, and FBXL5. However, FBXW5, KDM2A, and FBXO2 exhibit high expression levels in GC. Considering that each FBPs can target multiple substrates, their effects on tumorigenesis can be complex and dependent on the cellular context (**Table 1**).

Several studies have demonstrated that differential expression of FBPs is associated with poor prognosis in GC. Calcagno *et al.* reported that dysregulation of FBXW7 mRNA expression correlated with lymph node metastasis and advanced stages of GC, suggesting that FBXW7 may serve as an indicator of poor prognosis in GC 80. Similarly, altered expression of FBXW7 in the presence of P53 mutations was associated with poor prognosis in GC 81. Immunohistochemical analysis conducted by Li *et al.* showed that low FBXW7 expression in primary GC was associated with poorly differentiated tumor cells, shorter overall survival, and reduced response to adjuvant chemotherapy 82. Highlighted the impact of aberrant expression of FBPs on GC progression. Specifically, FBPs have an anticancer role in GC. A study by Kogure demonstrated that low FBXO45 expression was associated with increased cancer progression and poorer prognosis in GC patients 83. Similarly, low FBX8 expression was associated with shorter overall survival and poorer prognosis 71. In contrast, FBPs are also pro-cancer in GC, overexpression of FBXO11 in GC was associated with larger tumor size, lymph node metastasis, advanced TNM stage, and shorter survival 77.

High expression of FBXW5 was also correlated with poor prognosis 73. Interestingly, FBXO50 was found to be highly expressed in GC, and patients with high FBXO50 expression had a significantly higher prevalence of recurrence after curative gastrectomy and shorter overall survival 78. These findings suggest that differential expression of FBPs in GC not only promotes tumor progression but also inhibits it, emphasizing the dual role of FBPs in GC. Overall, FBPs play a significant role in the progression of GC.

## Relationship between F-box proteins and gastric cancer

Multiple events contribute to the malignant characteristics of cells, including sustained growth, resistance to cell death, induced invasion and metastasis, and increased resistance to chemotherapy 84. Mutations in oncogenes and tumor suppressor genes are characteristic of cancer. In recent years, FBPs have garnered attention for their crucial functions in mediating oncogenes and tumor suppressors in GC, thereby regulating various cancer-related features (**Table 2**).

### 3.1 F-box proteins are involved in the proliferation of GC

FBPs have been shown to regulate the growth and proliferation of GC cells. For example, FBXW7 mediates the degradation of GFI1, inhibiting the proliferation of GC cells 85, Similarly, overexpression of FBXL2 inhibits GC proliferation by degrading ubiquitinated fork head box M1 (FOXM1) transcription factor in GC cell lines 68. Knockdown of FBX8 significantly promotes the proliferation and invasion of BGC823 cells 71. In GC, Fbxo21 can inhibit proliferation, in part, by down-regulating Nr2f2 72. Additionally, FBPs can regulate proliferation by modulating other cell death modalities. Knockdown of KDM2B (FBXL10) immediately induces autophagy and subsequently inhibits GC cell proliferation 79. FBPs have been found to regulate GC proliferation through multiple distinct pathways, indicating their potential as critical upstream targets within the GC proliferation cascade.

### 3.2 F-box proteins are involved in invasion and metastasis of gastric cancer

**Figure 4:**
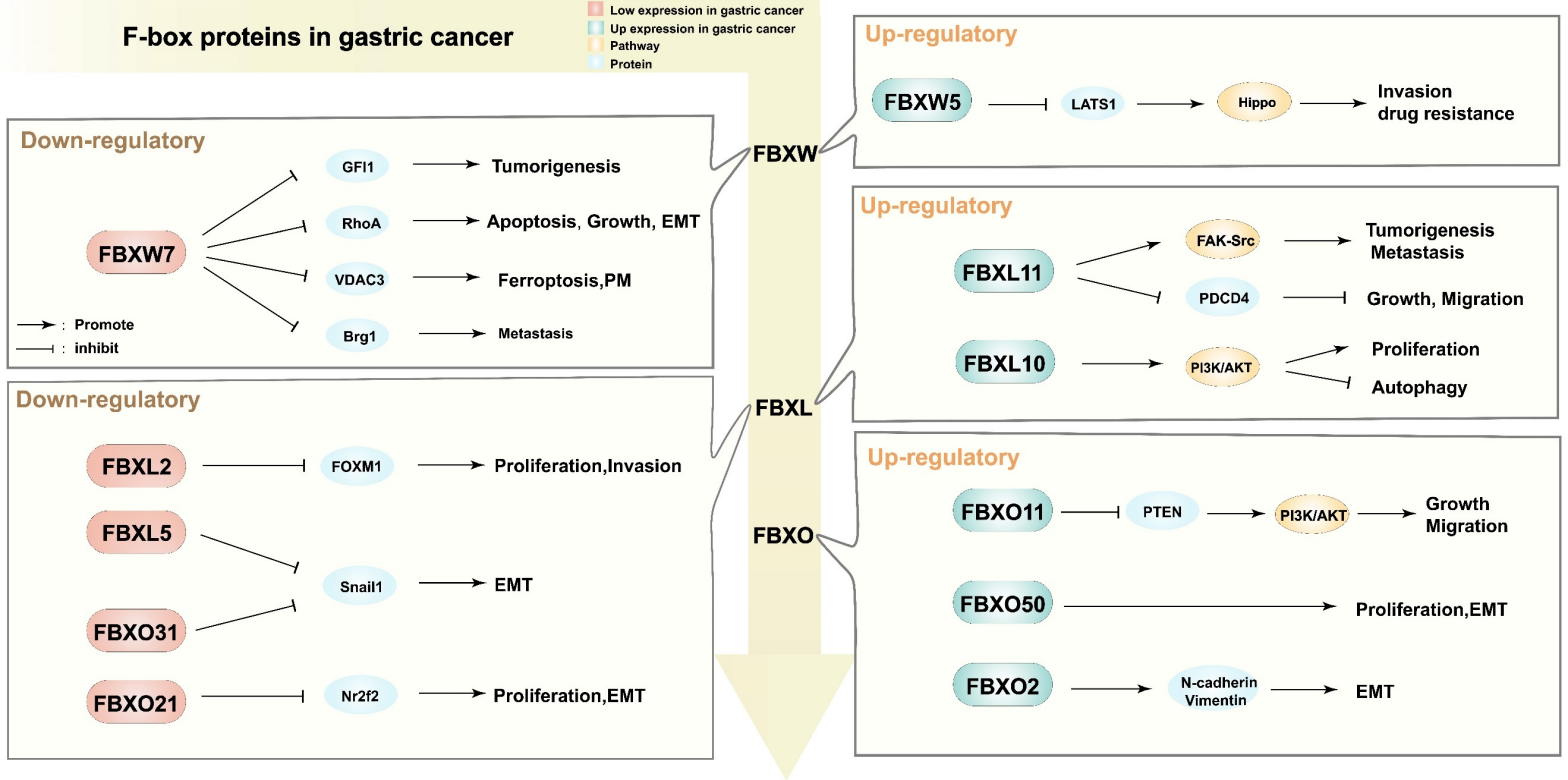
Different regulatory functions of F-box proteins in gastric cancer. PM: peritoneal metastasis; EMT: epithelial-mesenchymal transition; GFI1: Growth factor independence 1; RhoA: Ras homolog gene family member A; VDAC3: Voltage dependent anion channel 3; Brg1: Brahma related gene 1; FOXM1: Forkhead box M1; Snail1: snail family transcriptional repressor 1; Nr2f2: Nuclear receptor subfamily 2 group F member 2; LATS1: Large Tumor Suppressor Kinase 1; Hippo: Hippo signaling pathway; FAK-Src: focal adhesion kinase and c-Src signaling pathway; PDCD4: programmed cell death 4; PI3K/AKT: phosphatidylinositol 3-kinase/protein kinase B signaling pathway; PTEN: Phosphatase and tensin homolog; N-cadherin/Vimentin: the expression of interstitial markers.

Metastasis is a major contributor to the poor prognosis of GC. Several studies have demonstrated that FBPs can affect GC invasion and metastasis by controlling epithelial-mesenchymal transition (EMT) (**Figure 4**). FBXW7 downregulates the RhoA signaling pathway, inhibiting EMT in GC 66. FBXW7 mediates Brg1 degradation, thus inhibiting GC metastasis 86. FBXO21 inhibits EMT, in part, through down-regulating Nr2f2 72. FBXL5, FBXO31, and FBXW7 negatively regulate EMT-enhancing factors such as Snail 1 or ZEB 1, inhibiting GC metastasis 66, 69, 70. In a xenograft model of nude mice, FBX8 was found to be sufficient to inhibit metastasis 71. Moreover, FBPs can regulate GC metastasis and invasion through the modulation of signaling pathways. For instance, FBXW5 inactivates the Hippo signaling pathway by enhancing LATS1 ubiquitination and degradation, promoting GC cell invasion and metastasis 73. FBXW5 promotes tumorigenesis and metastasis in GC through activation of the FAK-Src signaling pathway 74. FBXO11 acts as an oncogene by suppressing PTEN and activating the PI3K/AKT pathway, thus promoting EMT in GC 77. Multiple FBPs have been shown to be highly correlated with metastatic invasion of GC, and different types of FBPs mediate metastatic invasion by different mechanisms, and those evidences support that FBPs are key upstream targets of EMT.

### 3.3 F-box proteins are involved in Chemoresistance of GC

Chemoresistance remains a major challenge in the treatment of advanced GC 87, 88. Understanding the molecular mechanisms underlying chemoresistance is crucial. FBXW7 has been identified as a tumor suppressor gene that reduces important oncoproteins, associated oncogenic effects, and cell cycle progression. Clinical data have shown that macrophage-derived exosomal miR-223 promotes doxorubicin resistance in GC cells by inhibiting FBXW7 89. Exosomal miR-500a-3p has been found to promote resistance to cisplatin and enhance stemness properties of GC cells by targeting FBXW7 90. Increased expression of miR-363 promotes cell proliferation and chemoresistance through direct targeting of the tumor suppressor FBXW7 91. FBXW7 has also been identified as a direct and functional target gene of miR-223, mediating DDP resistance in human GC 92. The miR-223/FBXW7 pathway has been shown to regulate the sensitivity of HER2-positive GC cell lines to trastuzumab 93. Other FBPs have also been implicated in chemoresistance in GC. Wu demonstrated that depletion of FBXL5 enhances cisplatin resistance in GC cells through ERK and p38 activation 94. Knockdown of FBXO32 enhances 5-FU cytotoxicity in GC cells that acquired prior resistance to 5-FU 95. Downregulation of FBXL7 by Aurora Kinase A (AURKA) inhibits Survivin degradation, leading to enhanced drug resistance 96 (**Table 3**). This demonstrates the potent role of FBPs in the drug resistance mechanism of GC, suggesting that FBPs could represent a novel mechanism of chemotherapy resistance in this malignancy. Hence, the pursuit of targeted drugs specifically designed to counteract FBPs or their synergistic utilization holds great promise as an avenue to enhance the present treatment paradigm for GC.

## 4. Key molecules regulating F-box proteins in gastric cancer

Numerous distinct non-coding RNA (ncRNA) sequences are abundantly present within cells. Initially regarded as mere "junk" transcription products, ncRNAs have emerged as functional regulatory molecules that orchestrate essential cellular processes encompassing chromatin remodeling, transcriptional regulation, post-transcriptional modification, and signal transduction. By participating in intricate networks, ncRNAs possess the ability to influence multiple molecular targets, thereby eliciting specific cellular responses and determining cellular fate 98, 99.

Consequently, ncRNAs assume pivotal roles as regulators of physiological programs during both normal development and disease states. Notably, ncRNA genes are increasingly being recognized as valuable therapeutic targets for cancer treatment 100, opening up new avenues for tumor diagnostics. Moreover, emerging evidence highlights the involvement of ncRNAs in governing the expression of FBPs in human cancers. Specifically, microRNAs (miRNAs), long non-coding RNAs (lncRNAs), and circular RNAs (circRNAs) have been implicated in the modulation of FBPs expression in malignant tumors (**Table 4**). This section focuses on elucidating the mechanisms by which miRNAs target FBPs and contribute to the pathogenesis and progression of GC. Additionally, we briefly outline the regulatory roles of lncRNAs, circRNAs, and other biomarkers in relation to FBPs in GC. These collective findings underscore the potential of targeting ncRNAs as a novel approach to regulate FBPs for anti-GC therapy.

### 4.1 The regulatory roles of miRNA on F-box proteins in gastric cancer

**Figure 5:**
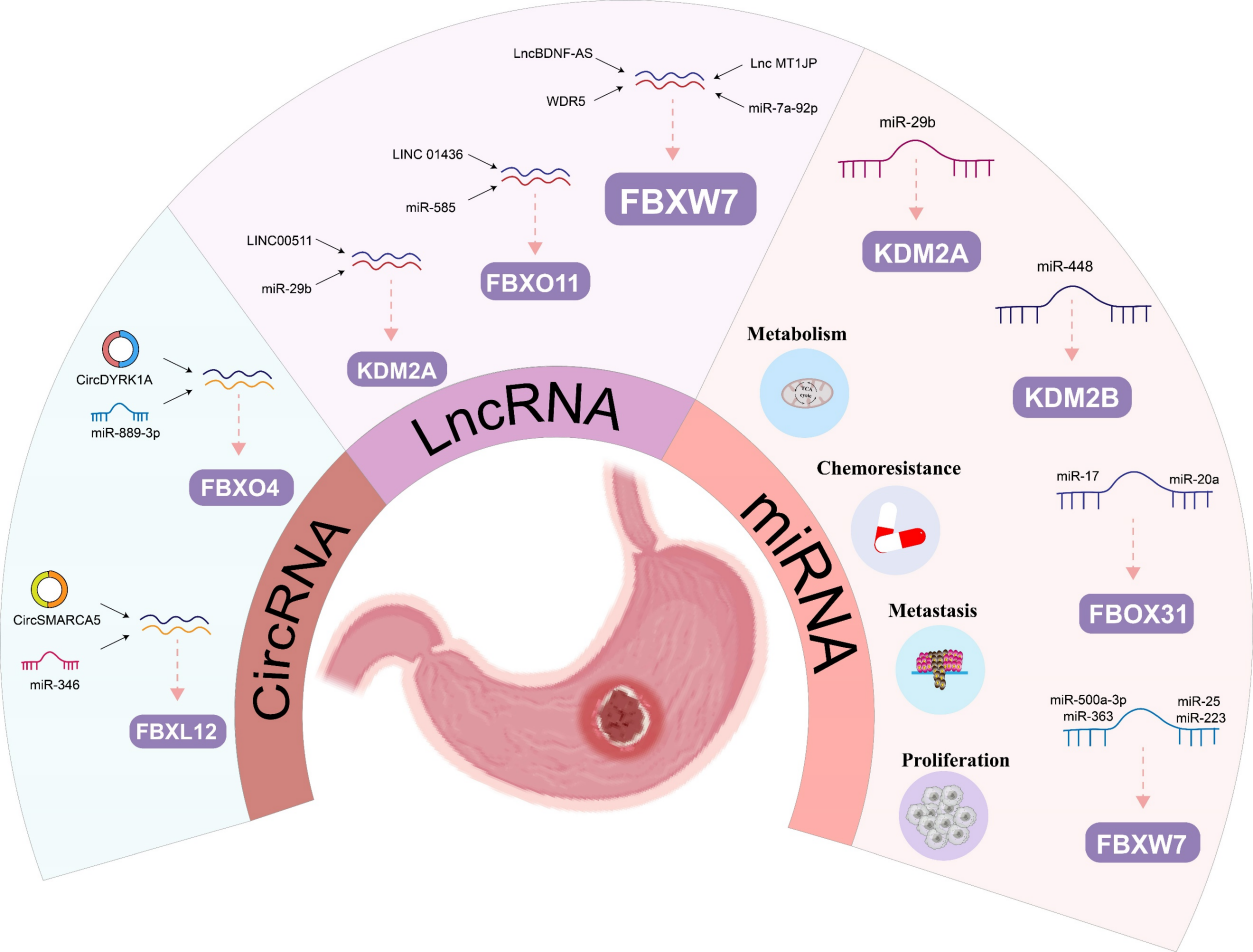
The regulatory roles of ncRNA on FBPs in gastric cancer: The regulatory roles of miRNA, lncRNAs and circRNAs on FBPs in gastric cancer.

MiRNAs have been increasingly recognized in recent years as regulatory genes that can bind mRNA through sequence complementation and inhibit protein translation and/or mRNA degradation, ultimately affecting human tumor progression and patient prognosis. The regulation of GC progression by mRNAs through FBPs is now gradually being demonstrated (**Figure 5**). Many studies showed *in vivo* and *in vitro* experiments that MiR-92a, miR-25 and miR-223 could promote the cell proliferation, invasion and migration through FBXW7 in GC 102, 104, 115. Meanwhile, MiR-25 could also have an antiapoptotic effect on GC by inhibiting FBXW7-promoting oncogenes, such as CCNE1 and MYC 103. FBXW7 also plays an important function in miRNA-mediated drug resistance in GC. Zhang *et al.* demonstrated that increased miR-363 expression was shown to promote GC proliferation and chemoresistance by directly targeting the tumor suppressor FBXW7^91^. Two studies showed that FBXW7 is a key target of MiR-500a-3p and miR-223 in mediating DDP resistance in GC 90, 92.

In addition to FBXW7, miRNA also regulates other members of the FBPs, for example, Zhang *et al.* found that miR-20a and miR-17 exerted pro-cancer effects by directly binding to the 3'-UTR of FBXO31 to inhibit FBXO31 expression 101. Furthermore, Hong *et al.* demonstrated that FBXL10 was the target of miR-448 that inhibited glycolysis and promoted oxidative phosphorylation 105. Ye et al identified that RUNX3 could mediate miR-29b up-regulation to inhibit the proliferation and migration of GC cells by targeting KDM2A 106. Thus, this growing evidence suggests that FBPs are key target protein for miRNAs regulating human GC.

### 4.2 The regulatory roles of lncRNAs on F-box proteins in gastric cancer

Besides the miRNAs mentioned in the appeal, other ncRNAs can also regulate FBPs (such as, lncRNAs and circRNAs) (**Figure 5**). LncRNAs play important roles in genomic transcription, translation, and post-translational modifications 116. Previous studies have shown that lncRNAs are involved in a variety of biological behaviors in GC, including proliferation, invasion, and metastasis 117-119. Huang *et al.* demonstrated that lncBDNF-AS can regulate FBXW7 expression by recruiting WDR5, thus affecting FBXW7 transcription, which regulates protein expression of VDAC3 through ubiquitination to protect GC cells from ferroptosis and promote the peritoneal metastasis (PM) 67. Zhang *et al.* found that higher lncRNA MT1JP was significantly associated with lymph node metastasis and progression, and MT1JP regulates GC progression by competitively binding to miR-92a-3p as a competitive endogenous RNA (ceRNA) and regulating FBXW7 expression 107. Similarly, another research team pointed out that LINC01436 promotes proliferation and metastasis of GC cells by regulating miR-585 and FBOX11 108. Furthermore, LINC00511/miR-29b/KDM2A axis could also be used as a diagnostic and therapeutic target for GC 109.

### 4.3 The regulatory roles of circRNAs on F-box proteins in gastric cancer

The expression and discovery of circRNA in tumors has become the latest research hotspot in the field of tumor RNA. Compared with traditional linear RNA, circRNA molecules have a closed-loop structure and are not affected by RNA exonucleases, and their expression is more stable and not easily degraded. Functionally, it mainly plays the role of miRNA sponge in the cell, which in turn relieves the repressive effect of miRNA on target genes and elevates the expression level of target genes 120. This provides a theoretical basis for circRNA regulation of FBPs (**Figure 5**). Li *et al.* confirmed that overexpression of circSMARCA5 inhibited GC cell proliferation, migration and invasion, mainly because circSMARCA5 could act as a miR-346 sponge that regulates the expression of FBXL2 110. Similarly, circDYRK1A could act as a miR-889-3p sponge to upregulate FBXO4 expression and inhibit glutamine metabolism in GC, thereby promoting its progression 111.

### 4.4 The regulatory roles of others on F-box proteins in gastric cancer

**Figure 6:**
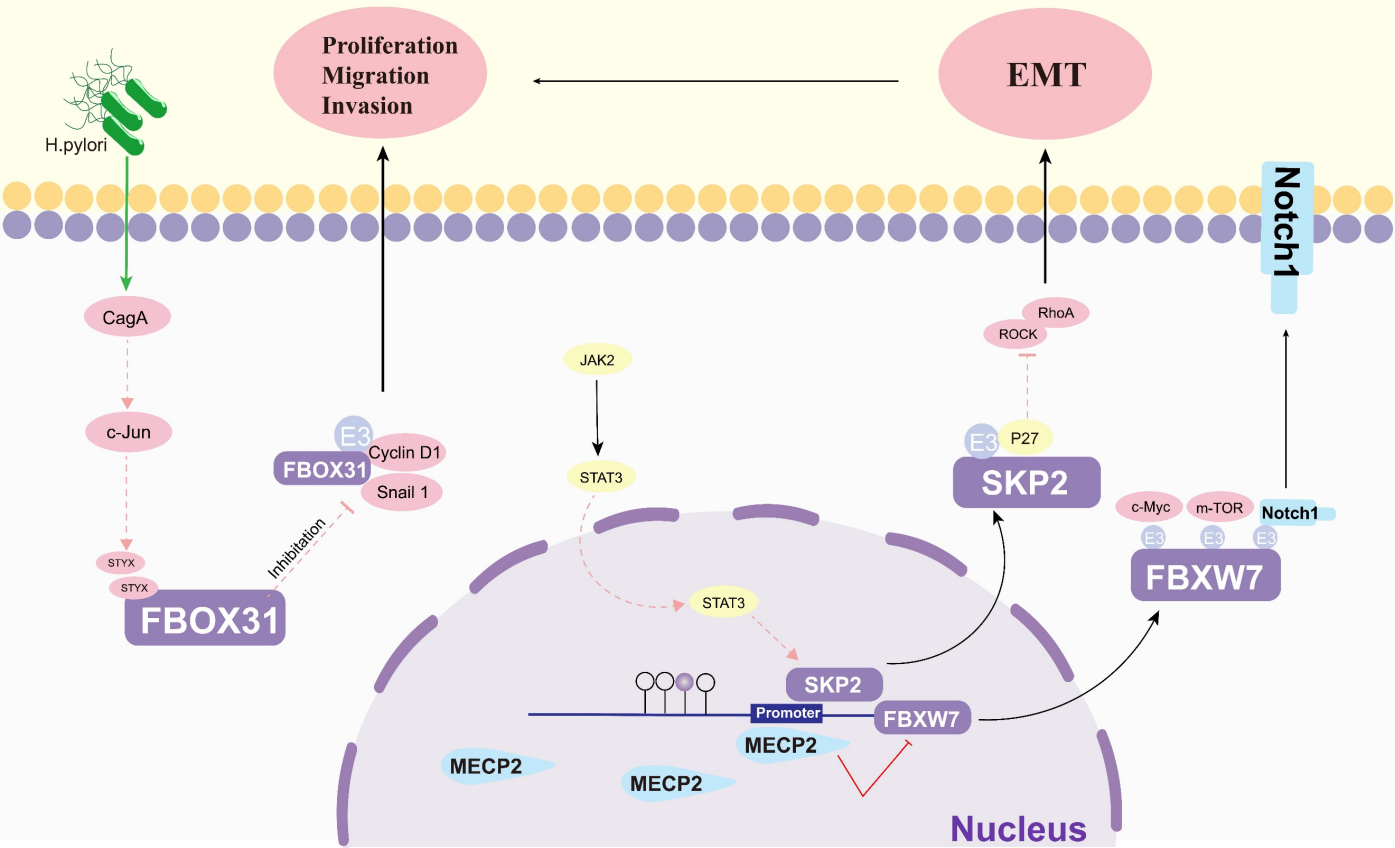
The regulatory roles of others on FBPs in gastric cancer. EMT: epithelial-mesenchymal transition; H. Pylori: helicobacter pylori; CagA: The cytotoxin-associated gene A; c-jun: AP-1 transcription Factor; STYX: Serine/threonine/tyrosine-interacting protein; Cyclin D1: A cell cycle protein; JAK2: Janus kinase 2; Snail1: snail family transcriptional repressor 1; STAT3: signal transducer and activator of transcription 3; SKP2: S-phase kinase-associated protein 2; MECP2: Methyl CpG binding protein 2; ROCK: Rho-associated coiled-coil forming protein kinase; RhoA: Ras homolog gene family member A; p27(SKIP1): The Cyclin-dependent kinase regulator; c-Myc: BHLH Transcription Factor; Notch1: Notch Homolog Protein 1.

Protein cancer biomarkers have multiple clinical purposes during disease progression, both in early and late stages (**Figure 6**). The search for new and better biomarkers has become an integral part of contemporary cancer research 121. Methyl-CpG-binding protein 2 (MECP2), an epigenetic regulatory factor, promotes the carcinogenesis and progression of a number of cancers. Zhao *et al.* found that MECP2 could regulate the Notch1/C-MYC/mTOR signaling pathway by inhibiting FBXW7 transcription, thereby promoting GC cell migration and invasion 114. The catalytically inactive pseudophosphatase serine/threonine/tyrosine interacting protein (STYX) is a member of the protein tyrosine phosphatase family. Lui *et al.* suggested that STYX plays an oncogenic role in GC mainly by inhibiting FBXO31. Interestingly, both transcription factor c-Jun and Helicobacter pylori (H. pylori) infection were found to enhance the expression of STYX in GC 112. This suggests that FBPs can be a key factor in the development of H. pylori-induced GC. Lei *et al.* showed that SerpinB5 was expressed at higher levels in GC tissues than in corresponding normal tissues and was associated with GC progression, and further studies revealed that KHDRBS 3 and FBXO32 are key molecules of SerpinB5 in GC carcinogenesis 122. One study found that the interaction between STAT3 and FBXL1(SKP2)/p27/p21 pathway - plays an important role in mediating the motility, migration and invasion of GC cells 113.

## 5. Prospects of the F-box proteins in the treatment of gastric cancer

The development of new drugs with specific targets for GC has always been an important part of oncology treatment, such as human epidermal growth factor receptor 2(HER-2) targeting agent Trastuzumab 123, vascular epidermal growth factor receptor (VEGFR) targeting agent Ramucirumab 124, 125, VEGFR-2 targeting agent Apatinib 126 and so on. It has been widely used in the treatment of GC patients, bringing enough benefits to GC patients. However, the existence of inter- and intra-patient heterogeneity, as well as poor efficacy and drug resistance, bring great challenges to targeted therapy, so it is crucial to develop new targeted drugs 127.

In GC as researchers have studied FBPs in depth, targeted blockers have now been developed for some key FBPs or related pathways, providing a theoretical basis for the development of targeted drugs for GC. Ueda *et al.* showed that O-GlcNAcase inhibitor Thiamet G (TMG) could promote GC progression by inhibiting FBXL2-mediated FOXM1 degradation 128. Wu *et al.* found that AICAR (an AMPK activator) could increase the expression of tumor suppressor genes FBXW7 and enhanced the pro-apoptotic effect of 5-FU in SGC-7901 cells 129. Soichiro *et al.* found that NS398 (a COX-2 inhibitor) induced inhibition of cell proliferation through cell cycle arrest and suppressed the expression of FBXL1 in COX-2-expressing GC cells 130. FBXL10 is normally expressed in GC, and downregulation of FBXL10 regulates the PI3K/AKT/mTOR signaling pathway to induce autophagy and subsequently inhibit proliferation, while the compound 3-methyladenine (3-MA), an inhibitor of autophagy, is able to reverse this process 79. BK697, a chemical inhibitor of FIRΔexon2, reversed the inhibitory effect of FIRΔexon2 on FBXW7 and inhibited progression in GC via the FBXW7/BRG1/Snai1 axis 131. Lycorine hydrochloride (LH), a derivative of lycorine, is an isoquinoline alkaloid extracted from lycoris^132^-^134^. Li *et al.* found that LH inhibits cell proliferation and induces apoptosis through promoting FBXW7-MCL1 axis in GC 135. These studies provide a new research direction for the development of small molecule drugs targeting FBPs in GC.

## 6. Discussion

In conclusion, this review provides convincing evidence for the role of the FBPs in GC progression. It shows the functional diversity of the FBPs. On the one hand some oncogenic members of the FBPs, which are down-regulated in GC, block the ubiquitination degradation process of some key oncogenic proteins and thus promote tumor metastasis invasion and drug resistance. On the other hand, some oncogenic FBPs are upregulated in GC and promote metastasis and invasion by activating some key oncogenic pathways (e.g. PI3K/Akt/mTOR). Thus, FBPs and their specific protein substrates may represent promising drug targets or biomarkers for GC, however, targeting FBPs remains challenging. Most previous studies have focused on the function of protein substrates of FBPs, and little is known about the regulatory role of FBPs or CRL itself in tumorigenesis. Moreover, most FBPs have multiple protein substrates, and some FBPs promote the degradation of oncoproteins and oncoproteins in GC, thus the function of FBPs is cellular environment dependent. A better understanding of the complex regulatory network of FBPs in GC, the involvement of their protein substrates and kinases in their post-translational modifications, and the mechanisms of cross-interaction with other signaling pathways are urgently needed for future studies.

Three E3-targeting small-molecule drugs, thalidomide, lenalidomide and pomalidomide, which bind a substrate receptor of the E3 ligase cereblon (CRBN)25, have been approved by the US Food and Drug Administration (FDA) 136. But with limited success in oncology treatment. Therefore, selective inhibitors targeting specific ubiquitin ligases and their protein substrates may be a better and more effective strategy for the treatment of GC. Growing evidence for modulation of FBPs and chemotherapy sensitivity by anticancer natural products 135. MLN4924 is an antitumor agent, Zhang *et al.* highlights the potential combination of MLN4924 and P27 inhibition to improve GC therapeutic efficacy 137. This suggests that develop natural products alone or in combination with modulators of FBPs and/or chemotherapeutic agents could show promising efficacy in human cancers, particularly drug-resistant cancers. However, further studies are necessary to identify the specific molecular targets of these natural products and to examine the efficacy and safety of these strategies in clinically relevant cancer models.

## Figures and Tables

**Table 1:** Expression of FBPs in GC.

FBPs	Expression in tissue	Sample size	Expression in cancer cells	Cancer cell lines	Relative normal cell lines	Ref.
FBXW7	Down	60	Down	AZ-521, MGC-803, BGC-823, SGC-7901	GES-1	66
	Down	66	Down	AGS, HGC-27, BGC-823, MGC-803, MKN-45	GES-1	67
FBXL2	down	15	down	NCI-N87	-	68
FBXL5	down	20	down	SNU-5, AGS	-	69
FBXO31	down	77	down	BGC-823, SGC-7901	-	70
FBX8	down	136	down	MGC-803, BGC-823, MKN45, AGS, SGC-7901	-	71
FBXO21	down	21	down	SGC-7901, BGC-823, MGC-803, MKN-45, MKN-28, AGS	GES-1	72
FBXW5	UP	16	UP	AGS, MKN-45, HGC-27, MGC-803, BGC-823, SGC-7901	GES-1	73
	-	-	UP	CLS145, MKN1, AGS, SNU1	-	74
FBXL11(KDM2A)	UP	61	UP	AGS, BGC 803, MGC-823, SGC 7901	GES-1	75
FBXO2	-	89	-	MGC-803, AGS, SGC-7901, MKN-28	-	76
FBXO11	Up	80	Up	SGC-7901, MGC-803, MKN-28, and BGC-823	GES-1	77
FBXO50	-	200	-	MKN1, MKN45, MKN74, NUGC2, NUGC3, NUGC4, SC-6-JCK, AGS, KATOIII, N87	-	78
FBXL10(KDM2B)	-	-	-	MKN-45, SGC-7901, N-87, HGC-27	GES-1	79

**Table 2:** *In vitro* functional characterization of FBPs in gastric cancer

FBPs	Substrate protein	Effect on viability/ proliferation	Effect on apoptosis	Effect on invasion/ metastasis	Reference
FBXW7	GFI1	inhibiting	-	-	85
FBXW7	Snail 1 / ZEB 1	inhibiting	promote	inhibiting	66
FBXW7	Brg1	-	-	inhibiting	86
FBXL2	FOXM1	inhibiting	-	inhibiting	68
FBXL5	Snail 1	-	-	inhibiting	69
FBXO31	Snail1	-	-	inhibiting	70
FBX8	-	inhibiting	-	inhibiting	71
FBXO21	Nr2f2	inhibiting	-	inhibiting	72
FBXW5	LATS1	promoting	-	promoting	73
FBXW5	-	-	-	promoting	74
KDM2A	-	promoting	-	promoting	75
FBXO2	-	-	-	promoting	76
FBXO11	PTEN	promoting	-	promoting	77
FBXO50	-	promoting	-	promoting	78
KDM2B	-	promoting	-	promoting	79

**Table 3:** The role of F-box protein in chemoresistance of gastric cancer

F-box protein	Substrate protein	Chemotherapeutic drugs	Effects on chemosensitivity	References
FBXW7	-	Cisplatin	Decreasing	92
	N-cadherin, vimentin	doxorubicin	Decreasing	89
	-	trastuzumab	Decreasing	93
	c-Myc/Mcl-1/cyclin E/c-Jun	DCF	Decreasing	91
	CD133, CD44 and SOX2	Cisplatin	Decreasing	90
FBXL5	RhoGDI2	Cisplatin	Decreasing	94
FBXO32	-	5-Fu	Decreasing	95
FBXL1	P27	Acyinom	Decreasing	97
FBXL7	survivin	doxorubicin	Decreasing	96

DCF: docetaxel + cisplatin + 5-fluorouracil; 5-Fu,5-fluorouracil

**Table 4:** Biomarkers targeting FBPs in gastric cancer

Biomarker	Expression in GC	Effects on FBP	FBPs	FBPs Functions	Reference
miR-20a/miR-17	Up	Inhibiting	FBXO31	suppressor	101
miR-25	Up	Inhibiting	FBXW7	suppressor	102
	Up	Inhibiting	FBXW7	suppressor	103
miR-223	Up	Inhibiting	FBXW7	suppressor	104
	Up	Inhibiting	FBXW7	-	89
	Up	Inhibiting	FBXW7	-	93
	Up	Inhibiting	FBXW7	-	92
miR-448	Up	Inhibiting	KDM2B	suppressor	105
miR-29b	Down	Inhibiting	KDM2A	suppressor	106
miR-363	Up	Inhibiting	FBXW7	-	91
miR-500a-3p	Up	Inhibiting	FBXW7	-	90
LncRNA MT1JP	Down	Inhibiting	FBXW7	suppressor	107
lncRNA BDNF-AS	Up	Inhibiting	FBXW7	suppressor	67
LINC 01436	Up	promoting	FBOX11	Promoter	108
LINC00511	Up	promoting	KDM2A	Promoter	109
circSMARCA5	Down	Inhibiting	FBXL2	suppressor	110
circDYRK1A	Down	Inhibiting	FBXO4	suppressor	111
STYX	Up	Inhibiting	FBXO31	suppressor	112
STAT3	Down	Inhibiting	FBXL1	Promoter	113
MECP2	Up	Inhibiting	FBXW7	suppressor	114

## Abbreviations

GC: gastric cancer
UPS: the ubiquitin-proteasome system
FBP: the F-box protein
SCF: the Skp1-Cullin1-F- box protein
ncRNAs: non-coding RNAs
E1s: ubiquitin-activating enzymes
E2s: ubiquitin-conjugating enzymes
E3s: ubiquitin ligases
DUBs: deubiquitinating enzymes
Ub: ubiquitin
Lys: lysine
Gly: glycine
RING: really interesting new gene
HECT: homologous to E6AP C-terminus
RBR: RING-in-between-RING
CRL: cullin-RING E3 ligase
APC/C: complex/cyclosome
SKP1: s-phase kinase-associated protein 1
ARID1A: AT-rich interactive domain 1A
EMT: epithelial-mesenchymal transition
LATS1/2: large tumor suppressor kinases 1/2
OS: overall survival
miRNA: microRNAs
lncRNA: long non-coding RNAs
circRNAs: circular RNAs
PM: peritoneal metastasis
DDP: cisplatin
DCF: docetaxel + cisplatin + 5-fluorouracil
5-Fu: 5-fluorouracil
UTRs: untranslated regions
ceRNA: competitive endogenous RNA
MECP2: methyl-CpG-binding protein 2
PM: peritoneal metastasis
LH: lycorine hydrochloride
STYX: serine/threonine/tyrosine interacting protein
H. Pylori: helicobacter pylori
Cul1: cullin1
RhoGDI2: Rho GDP dissociation inhibitor 2
AURKA: aurora Kinase A
HER-2: human epidermal growth factor receptor 2
VEGFR: vascular epidermal growth factor receptor
TMG: thiamet G
COX-2: cyclooxygenase -2
3-MA: 3-methyladenine
FDA: Food and Drug Administration
